# Contrast-enhanced computed tomography assessment of aortic stenosis

**DOI:** 10.1136/heartjnl-2020-318556

**Published:** 2021-01-29

**Authors:** Timothy RG Cartlidge, Rong Bing, Jacek Kwiecinski, Ezequiel Guzzetti, Tania A Pawade, Mhairi K Doris, Philip D Adamson, Daniele Massera, Maria Lembo, Frederique E C M Peeters, Christian Couture, Daniel S Berman, Damini Dey, Piotr Slomka, Philippe Pibarot, David E Newby, Marie-Annick Clavel, Marc R Dweck

**Affiliations:** 1 Centre for Cardiovascular Science, The University of Edinburgh, Edinburgh, UK; 2 Department of Interventional Cardiology and Angiology, Institute of Cardiology, Warsaw, Poland; 3 Quebec Heart and Lung Institute, Quebec City, Quebec, Canada; 4 Christchurch Heart Institute, University of Otago Christchurch, Christchurch, New Zealand; 5 Leon H Charney Division of Cardiology, New York University, New York City, New York, USA; 6 Department of Advanced Biomedical Sciences, University of Naples Federico II, Napoli, Italy; 7 Department of Imaging, Cedars-Sinai Medical Center, Los Angeles, California, USA; 8 Biomedical Imaging Research Institute, Cedars-Sinai Medical Center, Los Angeles, California, USA

**Keywords:** aortic valve stenosis, multidetector computed tomography

## Abstract

**Objectives:**

Non-contrast CT aortic valve calcium scoring ignores the contribution of valvular fibrosis in aortic stenosis. We assessed aortic valve calcific and non-calcific disease using contrast-enhanced CT.

**Methods:**

This was a post hoc analysis of 164 patients (median age 71 (IQR 66–77) years, 78% male) with aortic stenosis (41 mild, 89 moderate, 34 severe; 7% bicuspid) who underwent echocardiography and contrast-enhanced CT as part of imaging studies. Calcific and non-calcific (fibrosis) valve tissue volumes were quantified and indexed to annulus area, using Hounsfield unit thresholds calibrated against blood pool radiodensity. The fibrocalcific ratio assessed the relative contributions of valve fibrosis and calcification. The fibrocalcific volume (sum of indexed non-calcific and calcific volumes) was compared with aortic valve peak velocity and, in a subgroup, histology and valve weight.

**Results:**

Contrast-enhanced CT calcium volumes correlated with CT calcium score (r=0.80, p<0.001) and peak aortic jet velocity (r=0.55, p<0.001). The fibrocalcific ratio decreased with increasing aortic stenosis severity (mild: 1.29 (0.98–2.38), moderate: 0.87 (1.48–1.72), severe: 0.47 (0.33–0.78), p<0.001) while the fibrocalcific volume increased (mild: 109 (75–150), moderate: 191 (117–253), severe: 274 (213–344) mm^3^/cm^2^). Fibrocalcific volume correlated with ex vivo valve weight (r=0.72, p<0.001). Compared with the Agatston score, fibrocalcific volume demonstrated a better correlation with peak aortic jet velocity (r=0.59 and r=0.67, respectively), particularly in females (r=0.38 and r=0.72, respectively).

**Conclusions:**

Contrast-enhanced CT assessment of aortic valve calcific and non-calcific volumes correlates with aortic stenosis severity and may be preferable to non-contrast CT when fibrosis is a significant contributor to valve obstruction.

## Introduction

The pathogenesis of aortic stenosis involves an initiation phase characterised by mechanical injury, lipid deposition and a localised inflammatory response, followed by a propagation phase where progressive valve fibrosis and calcification promote worsening valvular stenosis.^1^ Recent guidelines recommend non-contrast CT calcium scoring of the aortic valve (CT-AVC) as an arbiter of aortic stenosis severity when echocardiographic measurements are discordant.^2^ This recommendation is based on data demonstrating the diagnostic accuracy of CT-AVC as a flow-independent measure and its correlation with disease progression and clinical events.^3–5^ However, CT-AVC has several important limitations. First, it offers little detail about valve morphology and is unable to localise the anatomical distribution of calcium in the valve and surrounding structures. Second, CT-AVC cannot quantify fibrosis, an important contributor to valve stenosis, and may therefore misclassify disease severity, particularly in young females and those with bicuspid valves.^6 7^ Third, it demonstrates only moderate associations with haemodynamic severity on echocardiography. Fourth, different thresholds for severe aortic stenosis have been proposed for males and females, with females consistently demonstrating lower calcium burden for a given degree of valvular stenosis, even after correction for body size.^3–5^

Contrast CT angiography is widely used to assess and quantify calcific and non-calcific plaques in the coronary vasculature.^8^ It is the gold standard method of anatomical assessment before transcatheter aortic valve intervention and is routinely incorporated into clinical workflows.^9 10^ In the present study, we used contrast-enhanced cardiac CT to evaluate aortic valve calcium volumes and also non-calcific leaflet thickening as a marker of valve fibrosis. We hypothesised that this technique would provide insights into the pathogenesis of aortic stenosis while accurately quantifying disease severity.

## Methods

### Study population

This was a post hoc analysis of patients with aortic stenosis (peak aortic jet velocity >2.5 m/s) from two study cohorts. The first cohort was the Study Investigating the Effect of Drugs Used to Treat Osteoporosis on the Progression of Aortic Stenosis (SALTIRE2) randomised controlled trial of novel drug treatments in aortic stenosis (NCT 02132026) which recruited individuals >50 years of age with aortic stenosis from the Edinburgh Heart Centre (online supplemental data). The second cohort comprised patients from the AVCa study (Quebec Heart and Lung Institute) in whom contrast-enhanced CT was performed <3 months prior to surgical aortic valve replacement (online supplemental data). A control group was selected from patients without aortic stenosis in the Dual Antiplatelet Therapy to Reduce Myocardial Injury (DIAMOND) trial (NCT 02110303), based on the lack of visual aortic valve calcification on CT.

10.1136/heartjnl-2020-318556.supp1 Supplementary data

### Study assessments and data collection

Clinical evaluation and echocardiography were undertaken as study-based procedures. CT-AVC and contrast-enhanced CT were performed in SALTIRE2 and DIAMOND, while contrast-enhanced CT scans, explanted aortic valve weights and histology were available in patients from AVCa.

### Image analysis

#### Doppler echocardiography

Echocardiographic assessment was performed using a standardised protocol.^11^ For the purposes of this post hoc analysis, aortic stenosis severity was categorised using peak aortic jet velocity (mild: 2.6–2.9 m/s, moderate: 3.0–4.0 m/s, severe: >4.0 m/s). Mean values were taken from three measurements when patients were in sinus rhythm and five measurements when in atrial fibrillation.

#### Computed tomography

Baseline CT imaging was performed on a 128-detector CT scanner (Biograph mCT, Siemens) using automated tube voltage modulation (CARE Dose 4D, 100–120 kV) or a 64-detector CT scanner (Somatom Definition, Siemens) with 0.75 mm slice thickness for contrast-enhanced CT or a tube voltage of 120 kV with 3 mm slice thickness for CT-AVC. ECG-gated CT-AVC and contrast-enhanced CT angiography were performed in diastole and breath held in expiration.

All CT analyses were performed blinded to echocardiographic assessments and vice versa. CT-AVC was quantified by an experienced operator (TP) using dedicated software (Vitrea Advanced, Vital Images, Minnetonka, USA).^5^ Analysis of contrast-enhanced CT was performed using OsiriX (V.8.0.3, Pixmeo SARL, Geneva, Switzerland). ECG-gated contrast-enhanced CT images were reconstructed in diastole. A multiplanar reconstruction was oriented in the short-axis plane of the aortic valve and, using the annulus as the reference slice, resliced in 3 mm increments through the valve.^12 13^ Slice thickness was selected to be consistent with CT-AVC. Blood pool contrast attenuation (Hounsfield units, HU) was sampled within a 2 cm^2^ circular region of interest in the centre of the aortic root lumen at the level of the sinotubular junction.

Quantification of aortic valve calcium volume and non-calcific leaflet volume was then performed using SliceOmatic (TomoVision, Magog, Canada). Using reoriented *en face* images, the region-growing tool was employed to select voxels within a defined range of attenuation values (online supplemental figure 1). The lower threshold for detection of calcium was 3 SDs above the mean attenuation measured within the blood pool region of interest. For non-calcific leaflet tissue, a fixed lower threshold of 30 HU was employed to exclude artefact (eg, photon starvation adjacent to dense calcification). The upper threshold for non-calcific tissue was calibrated to blood pool attenuation according to analysis of a derivation cohort comprising 100 patients’ scans. Two independent observers titrated the upper threshold for non-calcific leaflet thickening starting at 200 HU and adjusting by increments of 25 HU in either direction until the margins of the three aortic valve cusps were delineated without any highlighting of the blood pool. When this visually determined threshold (x) was plotted against mean blood pool attenuation (y), a strong linear relationship was identified (r^2^=0.90, p<0.001) with the equation x=41.46+0.42(y). Using these thresholds, areas of calcium and non-calcific leaflet thickening were assessed and quantified. Each adjacent 3 mm slice was assessed so that the whole volume of the valve was covered, with care to avoid regions of calcification in the aorta and coronary arteries.

#### Indexing to aortic annulus area

Leaflet volumes were indexed to the annulus area measured on contrast-enhanced CT. This method is widely used and repeatable.^9 14^ Valve weights were also indexed to the annulus area.

### Fibrocalcific ratio and fibrocalcific volume

The fibrocalcific ratio was derived by dividing the non-calcific by the calcific indexed leaflet volumes. A ratio >1.0 indicated a predominance of non-calcific (fibrotic) volume in the valve, while a ratio ≤1.0 indicated that ≥50% of the measured volume comprised calcification. The fibrocalcific volume was calculated by adding the indexed calcific and non-calcific leaflet volumes.

### Valve histology

In the AVCa cohort, aortic valves were excised at surgery, placed in a container filled with 4-(2-hydroxyethyl)-1-piperazineethanesulfonic acid (HEPES) solution and examined by a single pathologist who was blinded to the CT and echocardiographic assessments. One of the cusps was decalcified in Cal-Ex (Fisher, Nepean, Ontario, Canada) for 24 hours and fixed in formaldehyde 10% for histological processing. Aortic valve leaflets were embedded in optimal cutting temperature compound and 6 μm sections were obtained from a skilled operator using a cryotome. At least five histological sections per leaflet were analysed with Masson’s trichrome or Verhoeff-van Gieson staining to assess regions of fibrosis, calcification, thrombus or lipid. Semiquantitative assessment of valve fibrosis (1: mild, 2: moderate, 3: severe) and calcification (Warren-Yong score—1: absent, 2: mild valve thickening and early nodular calcification, 3: moderate thickening with many calcified nodules, 4: severe thickening with many calcified nodules) was performed according to previously published techniques.^7 15 16^ Valve cusps and any accompanying fragments were removed from HEPES solution, placed on absorbing paper and then weighed on laboratory scales with a tolerance of 0.01 g. Areas of non-calcific leaflet thickening observed on the CT were targeted for histological analysis.

### Statistical analysis

Baseline characteristics are reported as number (percentages) for categorical variables and mean±SD or median (IQR) for continuous variables as appropriate. Continuous variables were tested for normality with the Shapiro-Wilk test. One-way analysis of variance was used to compare continuous data across multiple variables. The χ^2^ test was used to compare categorical variables while the Student’s t-test or Wilcoxon rank sum test was used to compare continuous outcomes between two independent groups depending on whether they were normally distributed. Spearman's rank correlation coefficient was performed to assess the relationship between CT and echocardiographic measures. Linear regression models were constructed with peak aortic jet velocity as the dependent variable and age, female sex and either Agatston score or indexed fibrocalcific volume (both log2 transformed) as the dependent variables, with female sex and each of the latter as interaction terms. Interobserver variability was tested using the intraclass correlation coefficient (ICC; two-way random effects, agreement). Significance was taken at the two-sided 5% level (p<0.05). Statistical analysis was undertaken using R V.4.0.2 (Vienna, Austria).

## Results

A total of 164 participants with aortic stenosis (41 mild, 89 moderate, 34 severe) were included in the main analysis (table 1). The median age was 71 (66–77) years and the majority were male (78%). All patients had a left ventricular ejection fraction ≥50%. Peak aortic jet velocity was slightly higher in men (table 2), although this was not statistically significant. Four of 36 females were classified as severe aortic stenosis (11%) compared with 30 of 128 males (23%). Other baseline characteristics were similar between females and males. The time from echocardiography to CT was 0 (0–9) days. Thirty-eight participants (50% female, 67 (60–73) years) without evidence of aortic stenosis comprised the control group.

**Table 1:** Baseline demographics

	Overall n=164	Female n=36	Male n=128	P value
Age	71 (66–77)	71 (66–77)	71 (66–77)	0.820
Hypertension	116 (70.7)	25 (69.4)	91 (71.1)	0.999
Hypercholesterolaemia	95 (57.9)	22 (61.1)	73 (57.0)	0.752
Diabetes mellitus	43 (26.2)	8 (22.2)	35 (27.3)	0.705
Coronary artery disease	62 (37.8)	15 (41.7)	47 (36.7)	0.696
Current/ex-smoker	65 (39.6)	12 (33.3)	53 (41.4)	0.512
Medications				
Antiplatelets	92 (56.1)	16 (44.4)	76 (59.4)	0.146
Oral anticoagulation	17 (10.4)	3 (8.3)	14 (10.9)	0.153
ACE inhibitor/ARB	82 (50.0)	17 (47.2)	65 (50.8)	0.850
Beta blocker	61 (37.2)	11 (30.6)	50 (39.1)	0.462
Statin	107 (65.2)	19 (52.8)	88 (68.8)	0.092

ARB, angiotensin receptor blocker.

**Table 2:** Imaging parameters

	Overall n=164	Female n=36	Male n=128	P value
Echocardiography				
Peak aortic jet velocity (m/s)	3.44 (3.00–3.87)	3.26 (2.96–3.61)	3.53 (3.00–3.94)	0.106
Mean aortic valve gradient (mm Hg)	27 (18–34)	24 (18–32)	28 (19–34)	0.400
Aortic valve area (cm^2^)	1.01 (0.82–1.24)	1.03 (0.84–1.18)	1.01 (0.81–1.27)	0.735
CT				
Bicuspid aortic valve	11 (7)	0 (0)	11 (9)	0.120
Agatston score	1163 (670–2169)	460 (206–1010)	1474 (823–2362)	<0.001
Indexed contrast CT calcific volume (mm^3^/cm^2^)	82 (42–181)	33 (17–75)	103 (55–194)	<0.001
Indexed contrast non-calcific volume (mm^3^/cm^2^)	79 (49–104)	57 (39–98)	82 (56–104)	0.089
Indexed fibrocalcific volume (mm^3^/cm^2^)	187 (115–265)	115 (72–184)	203 (130–279)	<0.001
Fibrocalcific ratio	0.86 (0.47–1.54)	1.27 (0.78–4.23)	0.76 (0.41–1.45)	0.002
Blood pool radiodensity (HU)	401 (326–511)	550 (453–600)	366 (319–467)	<0.001

HU, Hounsfield units.

### Aortic valve calcification

**Figure 1:**
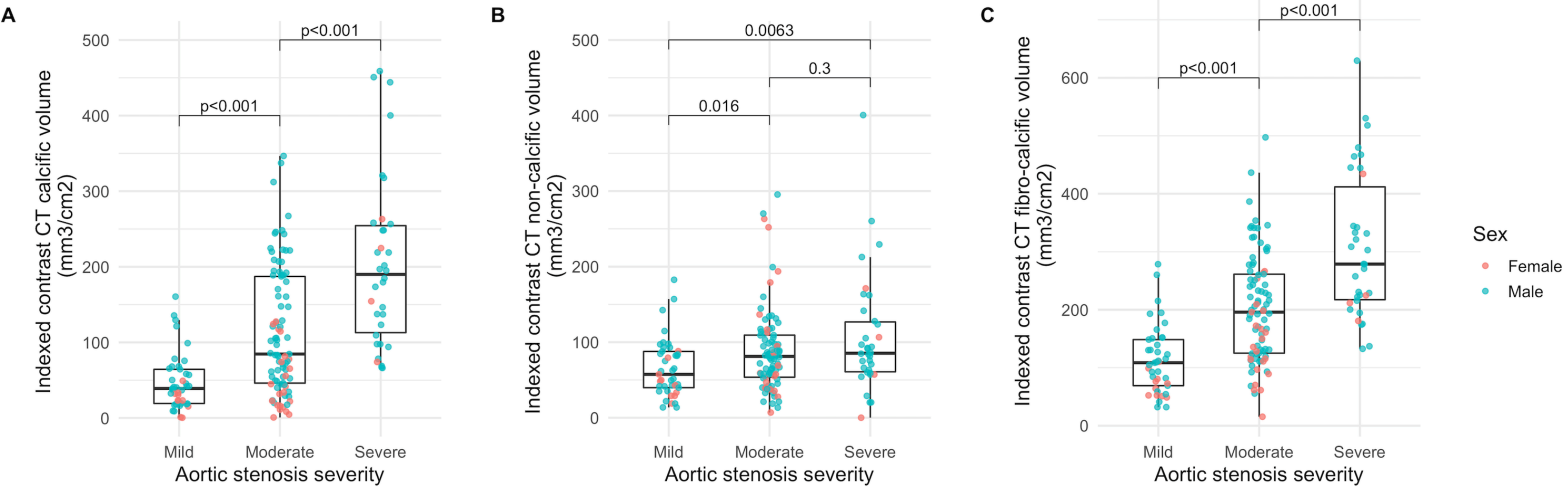
Indexed contrast CT leaflet volumes and aortic stenosis severity. Box plots of indexed contrast CT calcific (A), non-calcific (B), and fibrocalcific (C) volumes according to aortic stenosis severity. P values for Wilcoxon rank sum test.

In the control group, no aortic valve calcification was observed on either non-contrast or contrast-enhanced CT. In the aortic stenosis cohort, the indexed contrast CT calcium volume increased with stenosis severity (mild: 39 (19–64), moderate: 85 (46–187), severe: 190 (113–254) mm^3^/cm^2^; figure 1A) and correlated moderately well with peak aortic jet velocity (r=0.62, p<0.001; online supplemental table 1). There was excellent interobserver reproducibility (ICC: 0.95; 95% CI 0.88 to 0.98, p<0.001; online supplemental figure 2). Females had lower Agatston scores as well as indexed contrast CT calcium volumes compared with males (table 2).

### Non-calcific leaflet volume

**Figure 2:**
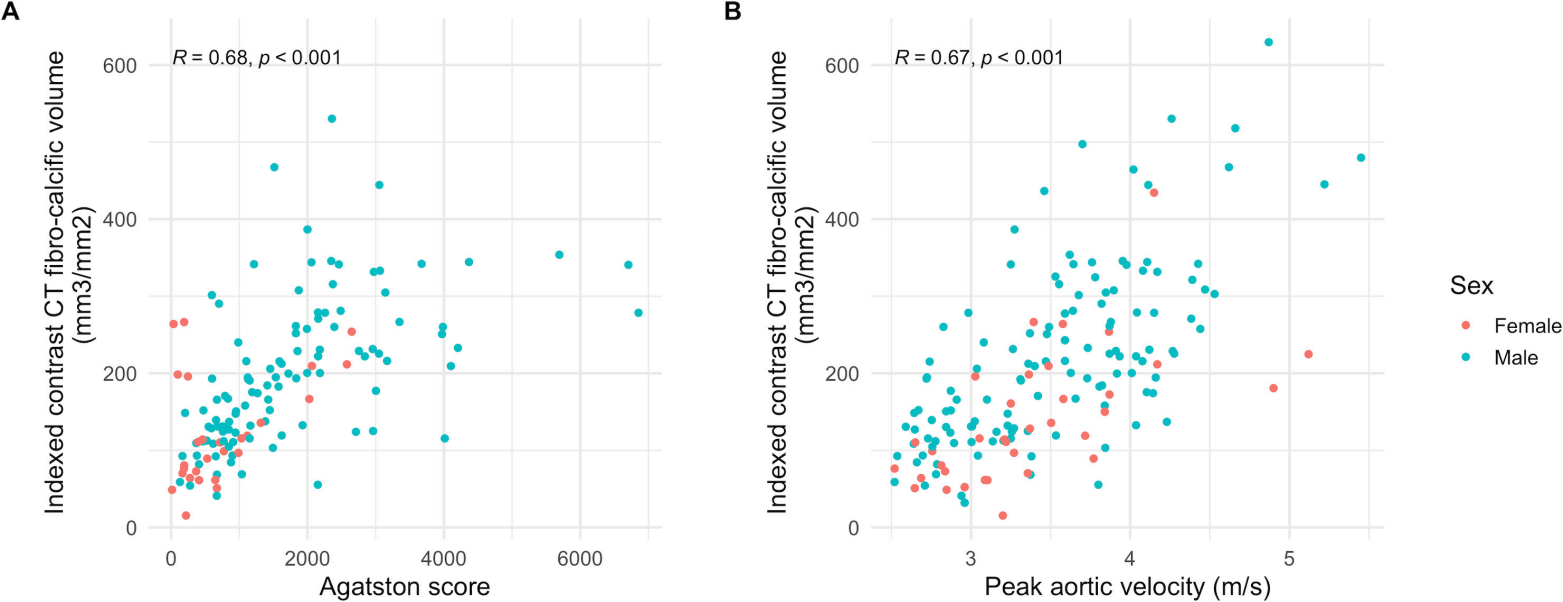
Fibrocalcific volume and standard measures of aortic stenosis severity. Scatter plots of indexed fibrocalcific volume, Agatston score (A) and peak aortic jet velocity (B). R and p values for Spearman's rank correlation coefficient.

In the control cohort, the indexed non-calcific leaflet volume was 30 (20–43) mm^3^/cm^2^ with no sex differences (females: 33 (19–43) vs males: 27 (21–39) mm^3^/cm^2^, p=0.18). In patients with aortic stenosis, indexed non-calcific leaflet volumes were higher than in the control cohort but only trended towards an increase with more severe stenosis (mild: 57 (40–88), moderate: 81 (54–109), severe: 85 (61–127) mm^3^/cm^2^; figure 2B). There was a weak correlation with peak velocity (r=0.27, p<0.001, online supplemental table 1). Interobserver reproducibility was excellent (ICC: 1.00; 95% CI 0.99 to 1.00, p<0.001; online supplemental figure 2). In contrast to the indexed calcific volume, the indexed non-calcific volume was only slightly lower in females than males (table 2).

### Fibrocalcific volume and fibrocalcific ratio

The indexed fibrocalcific volume increased across aortic stenosis severity categories (mild: 109 (69–148), moderate: 196 (125–261), severe: 279 (217–412) mm^3^/cm^2^; figure 1C). Compared with the Agatston score, fibrocalcific volume demonstrated a better correlation with peak aortic jet velocity (r=0.59 and r=0.67, respectively), particularly in females (r=0.38 and r=0.72, respectively) (table 3, figure 2A, B). The fibrocalcific ratio decreased with increasing stenosis severity (mild: 1.29 (0.90–2.30), moderate: 0.83 (0.47–1.57), severe: 0.52 (0.34–0.94)) and was higher in females than in males (online supplemental figure 3).

**Table 3:** Correlations between CT aortic valve assessments and echocardiography

	Peak aortic jet velocity	P value*
Agatston score†		
All	0.59	<0.001
Male	0.57	<0.001
Female	0.38	0.054
Indexed contrast CT fibrocalcific volume		
All	0.67	<0.001
Male	0.66	<0.001
Female	0.72	<0.001

*Spearman’s rank correlation coefficients.

†n=135/164.

On multivariable linear regression modelling with peak aortic jet velocity as the dependent variable and age, sex and Agatston score as independent variables, the latter two covariates were independently associated with peak velocity (p=0.001 and p<0.001, model adjusted r^2^=0.31), with an interaction between female sex and Agatston score (p=0.002; online supplemental table 2). When constructing the same model but replacing Agatston score with indexed fibrocalcific volume, the only independent predictor of peak aortic jet velocity was the indexed fibrocalcific volume (p<0.001, model adjusted r^2^=0.36). There was no interaction with female sex or difference in model fit on one-way analysis of variance (p=0.27).

### Histology

**Figure 3:**
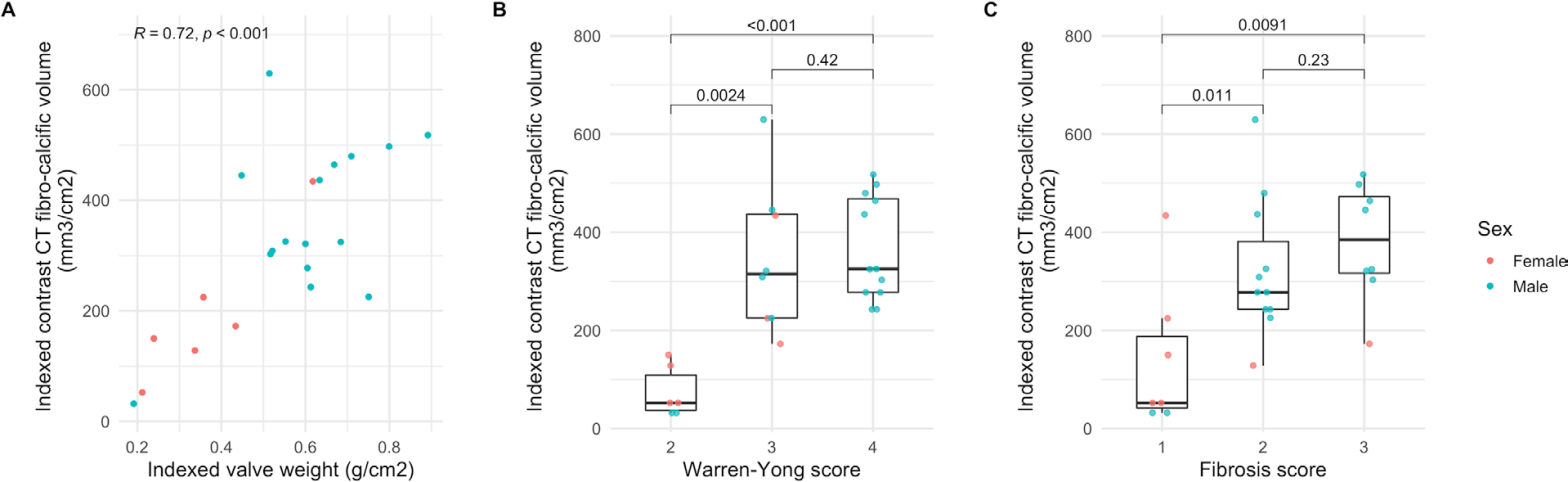
(A) Scatter plot of indexed valve weight and indexed fibrocalcific volume. (B) Box plots of indexed fibrocalcific volume and Warren-Yong score. (C) Box plots of indexed fibrocalcific volume and fibrosis score. P values for Spearman's rank correlation coefficient (A) and Wilcoxon rank sum test (B, C).

**Figure 4:**
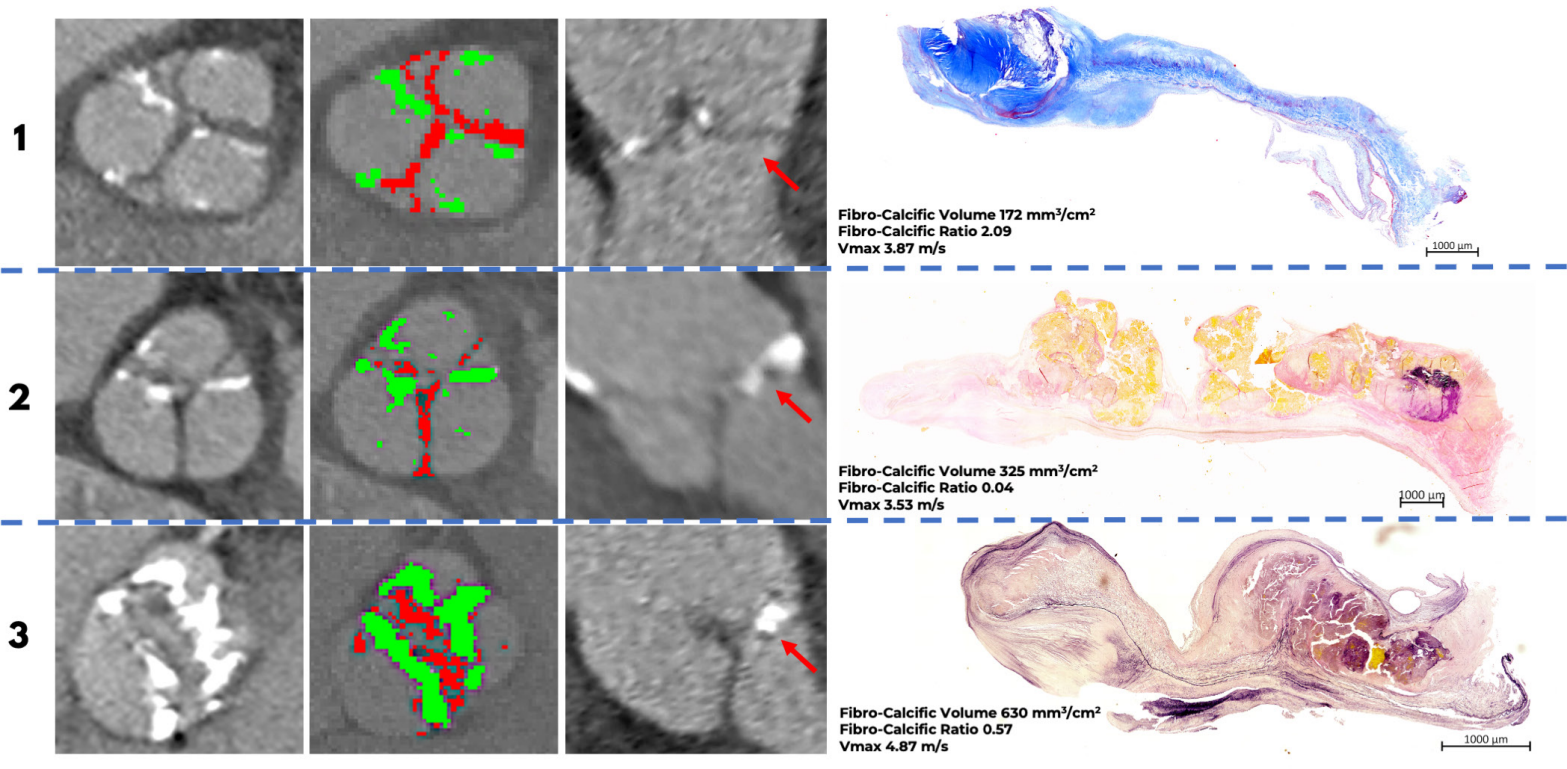
Contrast-enhanced CT and histology. Case 1: A tricuspid aortic valve from a woman with a large amount of fibrosis (red) compared with calcification (green) on CT. Histology confirms a preponderance of fibrosis in the valve consistent with the CT findings. There was no clear evidence of valve thrombosis or lipid infiltration. Red arrow denotes the leaflet corresponding to histology. Time from CT to surgery: 15 days. Masson’s trichrome staining: blue sections represent collagen; red/purple represents calcium. Case 2: A tricuspid aortic valve from a man with a small amount of fibrosis compared with calcification (from the three CT slices containing the aortic valve this one was the only one with significant fibrosis). This was again confirmed on histological analysis of the valve leaflet. Red arrow denotes the leaflet corresponding to histology. Time from CT to surgery: 15 days. Verhoeff-van Gieson staining: black represents elastic fibres, pink collagen fibres and yellow calcium. Case 3: A bicuspid aortic valve from a man with extensive fibrosis and calcification in the valve. Findings were again confirmed on histology with the spatial distribution of calcium and fibrosis on histology appearing similar to the calcific and non-calcific leaflet thickening on CT (Verhoeff-van Gieson staining). Red arrow denotes the leaflet corresponding to histology. Time from CT to surgery: 21 days.

Valve weight was available in 26 patients. There was a strong correlation between indexed fibrocalcific volume and indexed valve weight (figure 3A), in contrast to a probable weak correlation between peak aortic jet velocity and indexed valve weight (r=0.37, p=0.06). Histological examination confirmed the presence of valve fibrosis that spatially correlated with areas of non-calcific leaflet thickening on CT (figure 4). There was no thrombus or gross lipid deposition observed in these areas. The indexed fibrocalcific volume was higher in patients with higher Warren-Yong and fibrosis scores (figure 3B, C, online supplemental figure 4).

## Discussion

We describe a novel method of contrast-enhanced CT analysis that allows assessment of both aortic valve calcification and non-calcific leaflet thickening (fibrosis) in patients with aortic stenosis. We demonstrate the feasibility of contrast-enhanced CT assessment of aortic stenosis severity, with the indexed fibrocalcific volume correlating better with peak aortic jet velocity than CT-AVC in this cohort, particularly in females. Quantifying valve fibrosis, a facet of valvular disease that cannot be assessed with non-contrast CT, may be therefore important in some patients. Given the routine use of contrast-enhanced CT in clinical workflows for transcatheter aortic valve implantation, fibrocalcific volumes could be readily integrated into clinical practice, providing an alternative assessment for patients with uncertain disease severity.

Discordant echocardiographic measures of aortic stenosis severity are observed in around one-third of patients.^3 17^ CT-AVC has emerged as a useful tool in these patients, providing an anatomical, flow-independent assessment of disease severity that is well validated in concordant disease and supported by international guidelines. We found that assessments of valve calcification on contrast-enhanced CT correlated well with CT-AVC. However, progressive valve stiffening and narrowing in aortic stenosis occurs due to both fibrosis and calcification,^18 19^ and CT-AVC, which quantifies only the latter, may underestimate aortic stenosis severity in cases where valve obstruction is predominantly due to the former. Sex-specific CT-AVC thresholds for severe aortic stenosis may indirectly adjust for the increased valve fibrosis observed in females and generally offer good diagnostic accuracy compared with echocardiography.^5^ However, patients with severe aortic stenosis and concordant haemodynamic assessments of valve disease severity can have low Agatston scores.^6^ In addition, it can be challenging to differentiate valvular calcification from calcification of the aorta, left ventricular outflow tract, mitral valve or coronary arteries on axial non-contrast CT images. As such, there is a clear rationale for exploring alternative anatomical assessments of aortic stenosis that can overcome these issues.

We found the fibrocalcific volume to be a good measure of aortic stenosis severity that correlated well with echocardiographic measurements of valve haemodynamics and compared favourably with CT-AVC. Contrast-enhanced CT leaflet volumes also correlated with valve weight and semiquantitative histological assessments of calcification and fibrosis, although these findings are limited by our small sample size. Given the superior anatomical detail afforded by contrast-enhanced CT, calcific and non-calcific volumes also had excellent reproducibility.

The fibrocalcific ratio, a reflection of the relative contributions of non-calcific and calcific tissue to leaflet thickening, is highly variable. As an isolated measure, it is not necessarily useful in mildly or non-diseased aortic valves, where the ratio is infinite when calcium is absent. However, in our study, it is notable that although females—who comprised a minority of the cohort—had only slightly lower peak aortic jet velocities, their Agatston scores and indexed contrast CT calcific volumes were much lower than in males. Consequently, the fibrocalcific ratio was higher in females. It would therefore be congruent that the Agatston score correlated less well with peak aortic jet velocity in females. This finding is out of keeping with the larger evidence base, where the validation of CT-AVC was undertaken in populations with more severe disease.^5^ This is further emphasised by multivariable regression models in our cohort, which demonstrated that although Agatston score was independently associated with peak aortic jet velocity after adjusting for age and sex, there was an interaction between sex and Agatston score. Overall, we would suggest our findings do concur with existing data that demonstrate similar degrees of valve obstruction in females with lower valve calcium load, even indexed for body size. The poor performance of the Agatston score likely reflects the small sample size of females enrolled in this study, most of whom had non-severe aortic stenosis.

Our study has several strengths. It comprised patients prospectively recruited into studies across the spectrum of aortic stenosis severity, with systematic multimodality imaging assessments undertaken. The novel image analysis protocol proposes the use of flexible thresholds for defining calcific and non-calcific valve thickening based on contrast attenuation in the blood pool, and defines the aortic valve plane and annulus sizing in a uniform fashion that is well established. As a consequence, our technique is very reproducible. However, there are also some important limitations. The formula to set HU thresholds is derived, and as such will inevitably underestimate or overestimate non-calcific or calcific volumes in tissues that approach these thresholds, or where there is substantial variation in blood pool opacification. Accurate image analysis also requires adequate image quality and contrast opacification, and is challenging at the upper and lower extremes of blood pool contrast load. The majority of the cohort comprised patients with non-severe aortic stenosis, which differs from the derivation and validation cohorts for CT-AVC. A minority of the cohort were female and as such the sex differences observed must be interpreted with caution. Similarly, the number of patients with bicuspid valves—an important subgroup that differs from ‘degenerative’ aortic stenosis—was too limited to examine. We did not study patients with a low-flow state, which is a particular cohort where there is often diagnostic uncertainty. Consequently, the generalisability of this initial report is limited, and requires further validation in a larger cohort that comprised more females and more patients with severe aortic stenosis. Finally, our image analysis method is relatively time consuming, involving multiple steps and transfer of data between different software packages (~45 min per case). An integrated software solution to facilitate more rapid image analysis while maintaining accuracy and reproducibility is currently being investigated.

In conclusion, the aortic valve fibrocalcific volume as assessed by contrast-enhanced CT is an accurate and reproducible measure of aortic stenosis severity that quantifies both calcific and non-calcific leaflet volumes. As a promising tool for the assessment of aortic stenosis, the next steps are to improve efficiencies in image analysis, followed by larger scale validation to determine clinically useful thresholds.

**Key messages**

**What is already known on this subject?**

- Aortic valve calcium scoring using non-contrast CT is a measure of aortic stenosis severity that has been validated against echocardiography and is recommended as an arbiter of disease severity when echocardiography is equivocal. However, it provides poor anatomical definition and cannot assess non-calcific leaflet thickening, which may contribute to valvular obstruction.

**What might this study add?**

- We demonstrate the feasibility of using contrast-enhanced CT to quantify calcific and non-calcific valve leaflet thickening. Both the calcific leaflet volume and combined fibrocalcific leaflet volume correlated well with echocardiography, similar to non-contrast aortic valve calcium scoring. The addition of the non-calcific leaflet volume to the calcific leaflet volume particularly improved the correlation with echocardiography in females.

**How might this impact on clinical practice?**

- Our early data require wider validation, but there are clear potential clinical applications. The need for dedicated non-contrast CT as a measure of aortic stenosis severity may be obviated in patients already undergoing contrast CT—a standard assessment in the patients being considered for transcatheter aortic valve implantation, in whom a proportion may have discordant echocardiographic findings. Contrast-enhanced CT may also provide a more accurate anatomical assessment of stenosis severity in patients where leaflet fibrosis, rather than calcification, is a major contributor to valve obstruction.

## Data Availability

Data are available upon reasonable request.
