# Supplementary material for: Contrast-enhanced computed tomography assessment of aortic stenosis
Source: Heart. 2021 Jan 29;107(23):1905–11. doi: 10.1136/heartjnl-2020-318556 (PMC8600609; doi:10.1136/heartjnl-2020-318556)

SUPPLEMENTARY MATERIAL

Contrast-enhanced computed tomography assessment of aortic stenosis

Timothy RG Cartlidge MD[a*], Rong Bing MBBS[a*], Jacek Kwiecinski MD[b], Ezequiel Guzzetti MD[c], Tania A Pawade MD PhD[a], Mhairi K Doris MD[a], Philip D Adamson MD PhD[a,d], Daniele Massera MD[e], Maria Lembo MD[f], Frederique ECM Peeters MD[a], Christian Couture MD[c], Daniel S Berman MD[g], Damini Dey PhD[h], Piotr Slomka PhD[h], Philippe Pibarot PhD[c], David E Newby MD PhD[a], Marie-Annick Clavel DVM PhD[c], Marc R Dweck MD PhD[a]

[a] British Heart Foundation Centre for Cardiovascular Science, University of Edinburgh, Edinburgh, UK
[b] Department of Interventional Cardiology and Angiology, Institute of Cardiology, Warsaw, Poland
[c] Québec Heart & Lung Institute, Québec City, Canada
[d] Christchurch Heart Institute, University of Otago, Christchurch, New Zealand
[e] Leon H. Charney Division of Cardiology, New York University School of Medicine, New York, United States
[f] Department of Advanced Biomedical Sciences, Federico II University of Naples, Naples, Italy
[g] Cedars-Sinai Heart Institute, Los Angeles, California, USA
[h] Biomedical Imaging Research Institute, Cedars-Sinai Medical Center, Los Angeles, California, USA
* Authors contributed equally

METHODS

Study Patient Selection Criteria for SALTIRE 2

Inclusion Criteria

1. Age >50 years

2. Peak aortic jet velocity of >2.5 m/s on Doppler echocardiography

3. Grade 2-4 calcification of the aortic valve on echocardiography

Exclusion Criteria

- Anticipated or planned aortic valve surgery in the next 6 months
- Life expectancy <2 years
- Inability to undergo scanning
- Treatment for osteoporosis with bisphosphonates or denosumab
- Long-term corticosteroid use
- Abnormalities of the oesophagus or conditions, which delay oesophageal/gastric emptying
- Inability to sit or stand for at least 30 minutes
- Known allergy or intolerance to alendronate or denosumab, or any of their excipients
- Hypocalcaemia
- Regular calcium supplementation
- Dental extraction within 6 months
- History of osteonecrosis of the jaw
- Major or untreated cancers
- Poor dental hygiene

- Women of child-bearing potential who have experienced menarche, are pre-menopausal, have not been sterilised or who are currently pregnant
- Women who are breastfeeding
- Renal failure (estimated glomerular filtration rate of <30 mL/min)
- Allergy or contraindication to iodinated contrast
- Inability or unwilling to give informed consent
- Likelihood of non-compliance to treatment allocation or study protocol

Study Patient Selection Criteria for AVCa

<u>Inclusion Criteria</u>

1. Age >18 years
2. Being on the waiting list for a surgical aortic valve replacement

<u>Exclusion Criteria</u>

1. Non calcific aortic stenosis (i.e. rheumatic aortic stenosis, history of infective endocarditis, cervical or thoracic radiotherapy-induced valve lesions)
2. Reduced left ventricular ejection fraction (<50%)
3. Moderate-to-severe aortic regurgitation,
4. Previous aortic valve procedure (surgical repair, balloon valvuloplasty)

Supplementary Table 1 Correlations between contrast-enhanced computed tomography aortic valve assessments and echocardiography

		Peak aortic jet velocity	p*
**Indexed contrast CT calcific volume**	**All**	0.62	<0.001
	**Male**	0.61	<0.001
	**Female**	0.52	0.001
**Indexed contrast CT non-calcific volume**	**All**	0.27	<0.001
	**Male**	0.26	0.003
	**Female**	0.32	0.053

* Spearman's rank correlation coefficients.
CT: computed tomography

Supplementary Table 2 Multivariable linear regression models for prediction of peak aortic jet velocity

	Coefficient	P value	Adjusted R^2
**Model 1**			0.31
Intercept	0.47		
Age (per year)	0.00	0.97	
Sex (female)	0.86	0.001	
log2(Agatston score)	0.12	<0.001	
Sex*log2(Agatston score)	-0.09	0.002	
**Model 2***			0.36
Intercept	0.29		
Age (per year)	0.00	0.65	
Sex (female)	0.20	0.59	
log2(indexed contrast CT fibro-calcific volume)	0.19	<0.001	
Sex*log2(indexed contrast CT fibro-calcific volume)	-0.03	0.64	

*Restricted to same patients in Model 1, ie. those with Agatston score available.
CT: computed tomography

SUPPLEMENTARY FIGURE 1

Quantification of calcific and non-calcific tissue

A multiplanar reconstruction of the aortic valve is first created in Osirix as described in Methods. This reconstruction is then imported into SliceOmatic for quantification of leaflet volumes. The top left panel demonstrates a single slice through the valve, with the leaflet tips and valve calcification evident. Thresholds for calcification and non-calcific tissue are set as described in Methods. The region growing tool is then applied manually for calcific (top middle panel, green regions) and non-calcific (top right, red regions) tissue. An example of the software interface with both tissue components highlighted is shown in the bottom panel. These steps are repeated for each slice through the valve and the final volumes exported.

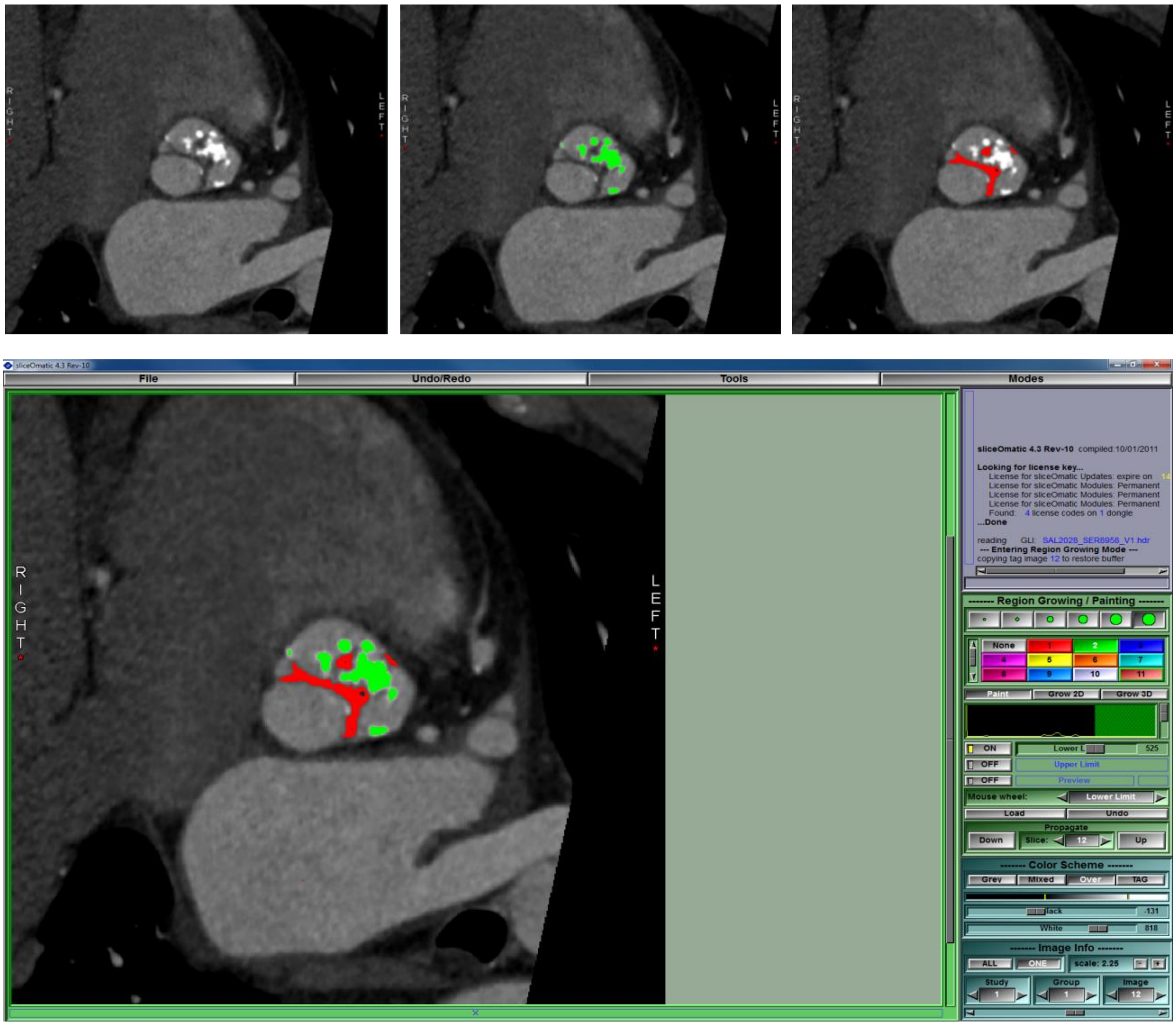

SUPPLEMENTARY FIGURE 2

Reproducibility

Measurements of non-calcific and calcific leaflet volumes were performed independently in 20 patients by two experienced operators (TC, JK) in order to assess inter-observer reproducibility. For this purpose, non-calcific and calcific volumes were not indexed to aortic valve area as the reproducibility of annular dimensions measured by CT angiography has been well described.

Inter-observer reproducibility of calcific and non-calcific volumes

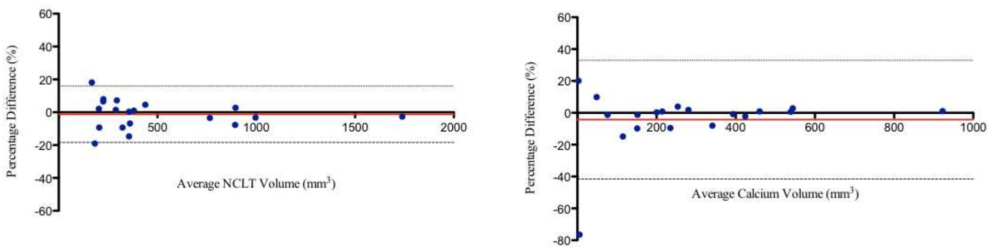

Bland-Altman plots of inter-observer reproducibility of non-calcific leaflet volume **(left)** (ICC (average measures): 0.997 (95% CI 0.992 to 0.999), p<0.001) and calcium volume **(right)** measured by contrast CT (ICC (average measures): 0.953 (95% CI 0.884 to 0.981), p<0.001).

SUPPLEMENTARY FIGURE 3

Indexed contrast CT leaflet volumes and sex

A: Box plots of fibro-calcific ratio according to sex. For visualization purposes only, 4 patients with high fibro-calcific ratios (356, 127, 74 and 42 mm^3/cm^2) have not been included in the plot. All 4 were female. Wilcoxon rank sum p<0.001. **B-D:** Box plots of indexed contrast CT fibro-calcific **(B)**, calcific **(C)** and non-calcific **(D)** volumes according to sex. P values for Wilcoxon rank sum test.

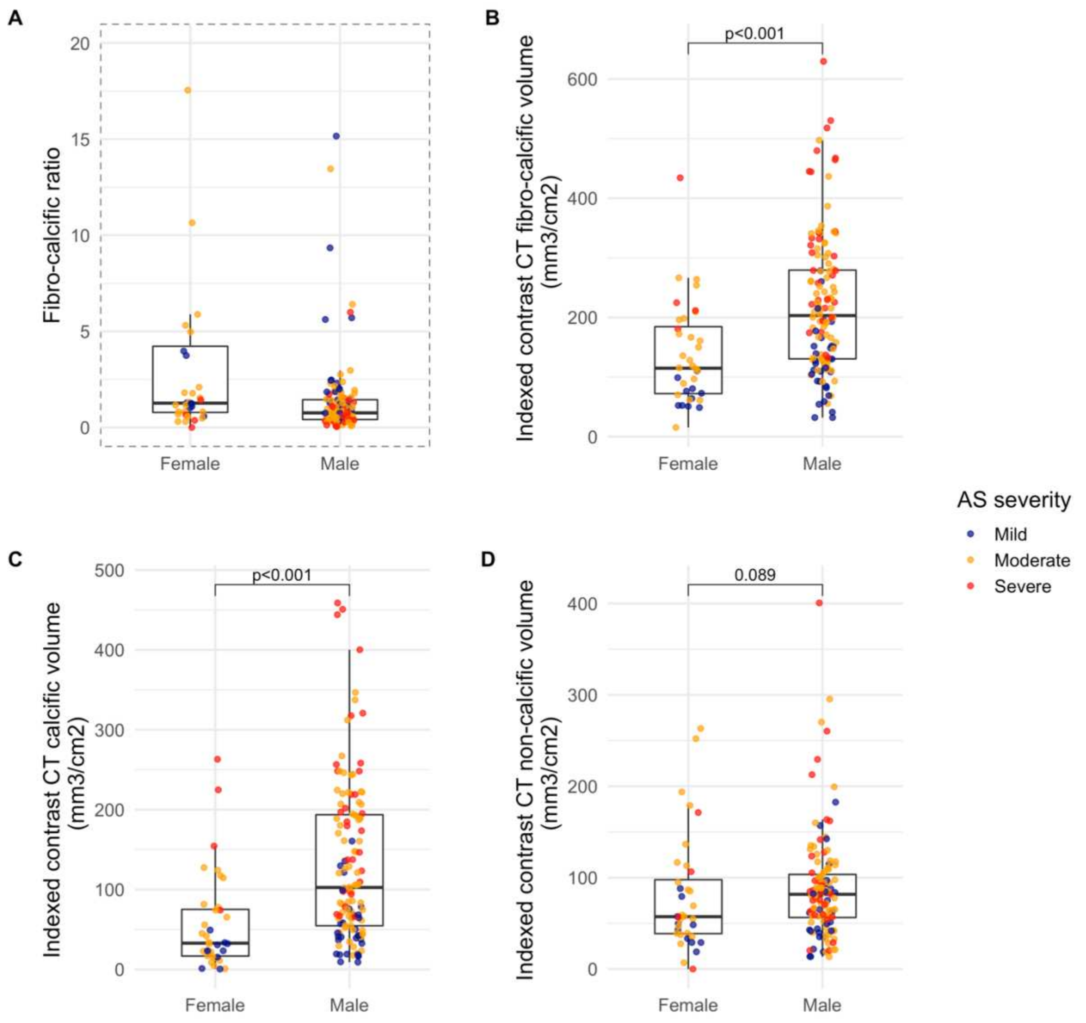

SUPPLEMENTARY FIGURE 4

Indexed contrast CT leaflet volumes and histology

A, B: Box plots of indexed contrast CT calcific and non-calcific volumes and Warren-Yong scores. **C, D:** Box plots of indexed contrast CT calcific and non-calcific volumes and fibrosis scores. P values for Wilcoxon rank sum test.

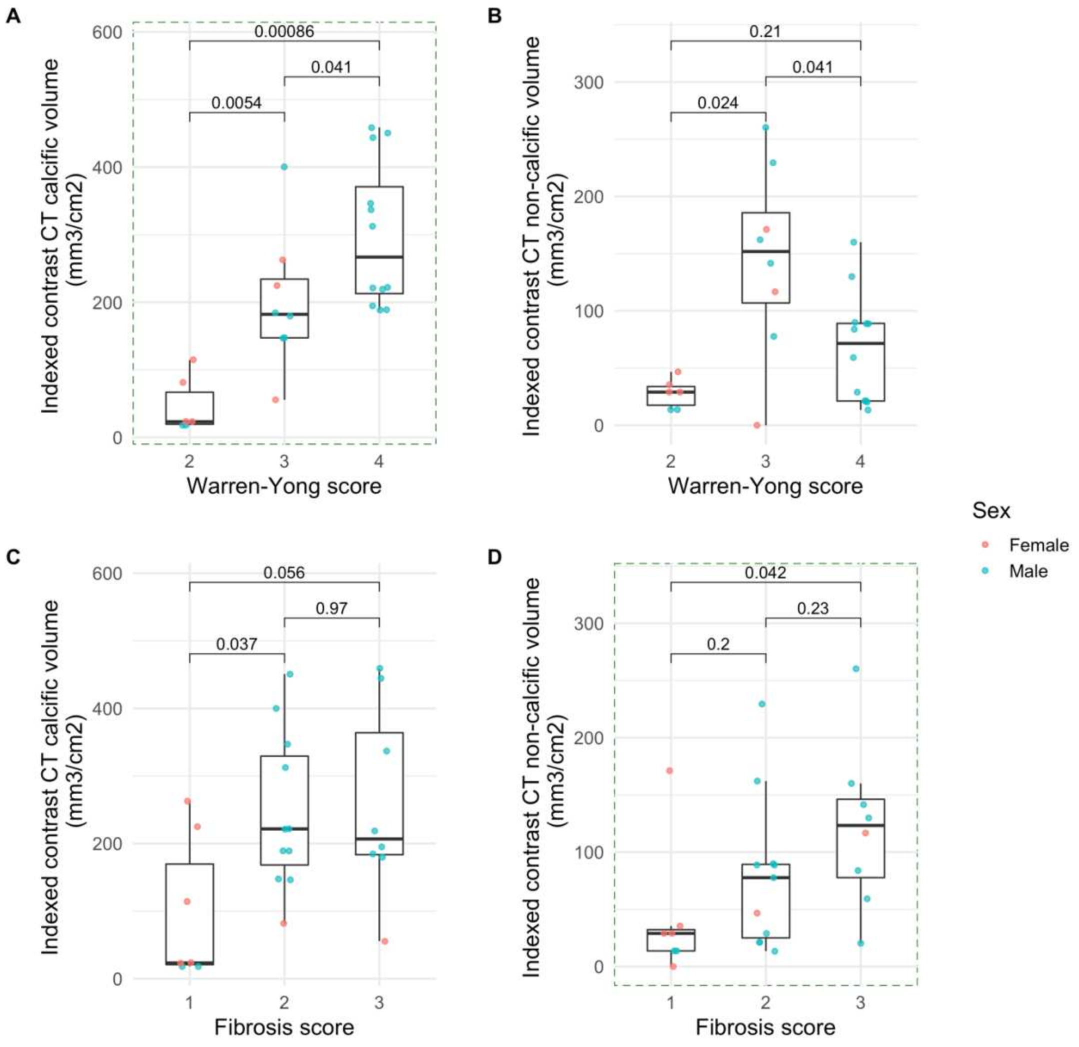

Supplement: Supplementary data [file heartjnl-2020-318556supp001.pdf]
